# Mother’s knowledge for environmental risks and self-awareness for the presence of pollutants in her living area in West and Central Anatolia: a cross-sectional survey

**DOI:** 10.1186/s12889-023-16684-7

**Published:** 2023-09-14

**Authors:** Sıddika Songül Yalçin, Gamze Gezgen Kesen, Bahar Güçiz Doğan, Suzan Yalçin, Songül Acar Vaizoğlu

**Affiliations:** 1 Department of Pediatrics, Faculty of Medicine, Hacettepe University, Ankara, Türkiye; 2 Department of Public Health, Faculty of Medicine, Hacettepe University, Ankara, Türkiye; 3 Department of Food Hygiene and Technology, Faculty of Veterinary Medicine, Selcuk University, Konya, Türkiye; 4 Department of Public Health, Faculty of Medicine, Near East University, Nicosia, Northern Cyprus

**Keywords:** Risk perception, Mothers, Environmental hazards

## Abstract

**Background:**

Environmental risk awareness is a key concept to raise awareness and plan future programs for environmental protection. A cross-sectional household survey aimed to find out the presence of environmental hazards next to living area and the mother’s knowledge levels about environmental risk factors with their related factors according to district development ranking, and Western and Central Anatolian regions with sampling from rural and urban residence.

**Method:**

The study was designed with household sampling weighted according to population density in 2008. Data on the demography and health status, dwelling characteristics of the residents are also collected in 2009. In addition, open-ended questions "What does environmental risk/hazard mean?" and "Which environmental risks/hazards are present in your environment?" were asked. The data collected from the survey were analyzed using multivariate binary logistic regression.

**Results:**

The sample included 3489 mothers living either in urban or rural areas. Of the mothers, 19.3% did not know what an environmental risk is and 75.7% stated that there was at least one environmental pollutant in their environment. The most commonly perceived risk factor was air pollution (23.0%), which was reported to be present in their living areas by 12.4%. Regions, residence, settlement features of the house, and health status of family members were associated with the perception of environmental risk at a statistically significant level.

**Conclusion:**

The neighborhood conditions and health status of family associated with the mother’s awareness for environmental risk factors. Communication and cooperation between local governments, health institutions, non-governmental organizations and other stakeholders should be strengthened to increase risk awareness.

## Introduction

 Chemical and physical environmental hazards such as air pollution, toxic chemicals (toxic elements, polycyclic aromatic hydrocarbons, pesticides, polychlorinated biphenyls, phthalates, bisphenols etc.), radiation, and stress during perinatal and childhood period are shown to be associated with fetal, childhood and adulthood disorders including birth defects, developmental disabilities, behavioral problems, metabolic disorders (obesity, endocrine disruption), and malignancies [1–12]. Notably, the foundation for these health impacts is laid during fetal and early childhood period, signifying the criticality of this developmental window. These effects can occur at doses much lower than those that affect adult health [3, 7]. As mothers serve as the primary conduits between their offspring and the external environment, their exposures to various chemicals and pollutants prior to or during pregnancy can significantly impact fetal and infant development [4, 7]. In addition, after the pregnancy period, the role of motherhood becomes important as mothers take on the responsibility of protecting their children from the potential negative effects of environmental hazards [13–16]. Herein, their actions, awareness, and protective measures during pregnancy and childhood can profoundly shape the health trajectories of their offspring [13]. Similarly, mothers having neither food safety-related risk perceptions nor protective behaviors showed significantly higher children’ hair Hg concentrations compared to counterparts [17]. Thus, recognizing the role of mothers as custodians of their children's well-being underscores the urgency of empowering them with information and resources to make informed choices that can ameliorate the potential long-term health consequences of environmental exposure [13]. On the other hand, the perceptual acuity and conscientious awareness of parents regarding the intricate landscape of environmental pollution, and their cognitive grasp of environmental challenges carry a pivotal role, resonating far beyond the boundaries of their immediate progeny and personal health with broader societal dimensions. This nuanced awareness engenders a proactive stance, wherein parents become instrumental agents in the preservation of ecological integrity and the mitigation of adverse anthropogenic impacts. Understanding and promoting maternal environmental risk awareness holds paramount significance in safeguarding the optimal development of the fetus, infant and young children. Moreover, as parents serve as the nucleus of foundational societal units, their informed decisions and conscientious behaviors permeate through social strata, initiating a ripple effect that reverberates throughout the community [14].

Risk perception is a fundamental cognitive process that influences how individuals perceive and respond to potential hazards and uncertainties in their environment. It plays a crucial role in shaping the decisions, behaviors, and attitudes towards various aspects of life and in comprehending how the public evaluates risks, shapes behaviors, and facilitates effective risk communication [18]. The perception and tolerance of risk are reported to be influenced by various factors, including individual characteristics, cultural and social influences, and the context in which risks are encountered [19]. The factors that influence individuals' risk perception are wide-ranging, encompassing not only their knowledge and comprehension of risk characteristics but also personal experiences, emotions, and the cultural, economic, and political contexts in which they exist [20, 21]. Also experts' objective assessment of risk differs significantly from the subjective judgment of non-experts [22]. The acceptance of risk by individuals is directly influenced by their intuitive perception. The first step in taking precautions for risk is to increase risk awareness by conducting risk perception studies specific to regions by evaluating the situations that relate this perception, such as environmental exposure, socioeconomic status, education level, previous expressions, etc. In a study on risk perception of food additives in China, considering consumers' cognitive rules and concerns, enhanced the effectiveness of knowledge dissemination about food additives compared to a traditional comprehensive-knowledge-based approach [22]. Risk perception refers to an individual's capability to recognize and evaluate the presence of a particular risk, while risk tolerance refers to a person's willingness to accept a specific level of risk. Although distinct, these concepts are closely interconnected. Several theories suggest that an inaccurate perception of risk can contribute to elevated levels of risk tolerance, potentially leading individuals to engage in high-risk behaviors [19]. According to protection motivation theory, the risk perception and use of personal protective measures increase when person has the reason of consern, oftentimes due to previous experiences [19].

Understanding and determining people's risk perception is the first step in raising public awareness and enabling people to make the right decision [23–25]. There is only one study evaluating risk perception of mothers in one province of Türkiye [26]. In that study, risk perception of mothers was found to be associated with rural–urban residence and presence of health problem. Mothers are a very important group since their knowledge and attitude are important to preserve the well-being and the environment for the future generations [14, 26–29]. When considering in national aspect, risk perception might change with district developmental index, regional living area and dwelling environmental characteristics. There is no study evaluating environmental risk perceptions in national level in world. From this point, we aimed to investigate the presence of environmental risk factors in living areas of families and the knowledge of mothers about environmental risks and the factors associated with risk awareness of mothers in West Anatolia and Central Anatolia, and in rural and urban residences, and district development ranking in Türkiye. With the results of the study, understanding the risk perception according to both the living area and presence of health problem will guide to inform the mothers about the protection of their children against environmental threats.

## Methods

### Study design

**Fig. 1:**
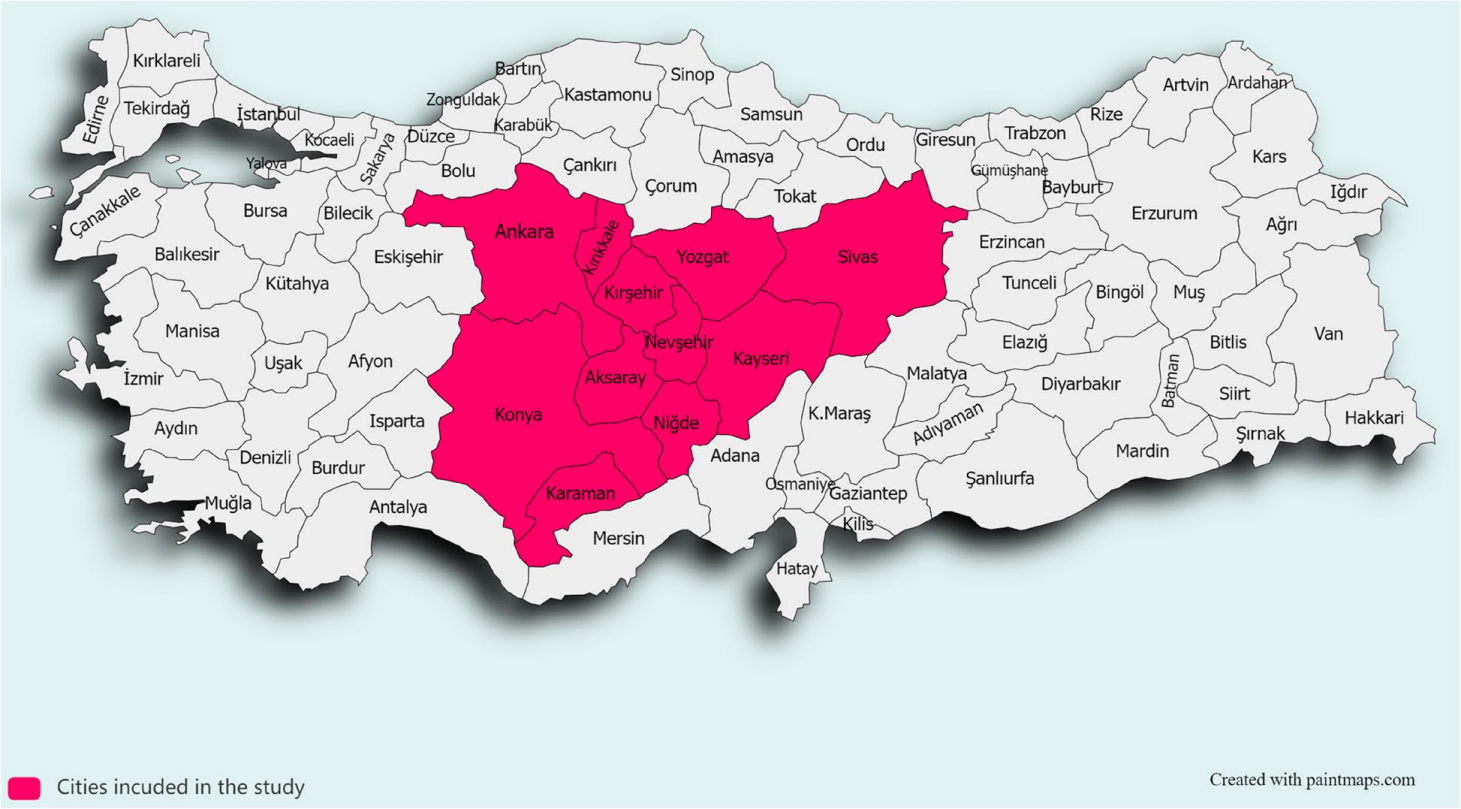
West and Central Anatolia regions with 11 cities in TÜRKİYE

This population-based cross-sectional household survey was conducted in two Nomenclature of Territorial Units for Statistics (NUTS) regions of Türkiye, consisting 11 provinces; Ankara, Konya, Karaman in West Anatolia region, Kayseri, Kırşehir, Nevşehir, Niğde, Sivas, Yozgat, Aksaray and Kırıkkale in Central Anatolia region (Fig. 1) [30]. Data collection was carried out between November 2008 and December 2009.

### Eligibility criteria

Mothers with at least one child aged 8-years were included in the study. Mothers will be the most knowledgeable when children in this age group are in primary school 2–3, positive health habits are taught in classes, children are dependent on their mothers for both their lessons and care, and mothers are counselled about risks during health follow-ups from pregnancy to childhood [31].

### Permissions

Hacettepe University Ethics Board of Non-Interventional Clinical Research approved the protocol. General permission for the survey was obtained from the Ministry of Health in Türkiye. The permission of the Public Security Department of the General Directorate of Security for providing safety to the interviewers and nurses working during the data collection period was obtained. The mothers were informed about the study and their written consent was obtained.

### Study size and household selection

The sample size was determined as 3142 participants in order to detect a condition with a prevalence of 5% (problems in terms of public health) with a 99% confidence interval, and a sampling error of d = 0.01 [32]. Considering 15% proportion of incomplete forms, ultimately, it was planned to reach 3613 households.

Household sample, weighted according to population density and rural–urban residence, was obtained from the Turkish Statistical Institute. Local Provincial Health Directorates and Health Centers / Community Health Centers were also informed about the study.

### Survey

The survey was carried out through face-to-face interviews with the mothers. The "Environmental History Form” was used to assess the environmental impact of the child's health problems [26, 33]. With this form, data related to the variables such as; 1) parental age, parental job, parental illness, sibling death history, smoking exposure, any ill person in the household, 2) house characteristics; heating source, domestic chemical use (pesticide, paint, hobbies, etc.), 3) the proximity of living area to the environmental risk factors including main roads, agricultural areas, animal shelters, construction sites, dumpster areas, swamps or puddles, rivers, streams, high voltage areas, base station, transformer sites, and factory or industrial zones.

Following The "Environmental History Form", two open-ended questions were asked: "What does environmental risk/hazard mean?" and "Which environmental risks/hazards are present in your environment?". The answers were classified by four researchers independently and cross checked afterwards as in the previous study [26]. The risks were classified as “security”, “air pollution”, “environmental pollution”, “municipality service problems”, “risk of accidents”, “contagious diseases”, “radiation- electromagnetic field (EMF)”, “illness/death”, “disasters”, “food-water pollution”, “chemicals”, “future anxiety”, “noise pollution” and “global warming”.

Professionals possessing specialized training in the field of survey administration were deployed to the residences of the mothers to execute the survey protocol. These trained personnel were equipped with the requisite expertise to proficiently administer the survey instruments and gather data in a meticulous manner. This approach underscores the meticulousness and precision with which the data collection process was undertaken, thereby enhancing the credibility and robustness of the ensuing dataset.

The areas where the families live was classified according to the district development ranking in 2022 [34]. The "District Development Ranking Survey (SEGE) 2022" conducted by the Ministry of Industry and Technology in Türkiye includes a comprehensive assessment of the socio-economic status of 973 districts. This assessment is based on the analysis of 56 variables covering critical dimensions such as demographics, employment, education, health, finance, competitiveness, innovation and quality of life. Each region was given a score based on composite variables to reflect relevant socio-economic progress. A total of six different levels of development have been created, including regions with superior scores in the premier tier, while relegating districts with lesser scores to subsequent tiers. This multidimensional assessment provides both a comprehensive understanding of regional developmental disparities and a fundamental framework for the formulation of targeted policies and informed decision-making processes [34]. In the present survey, 1555 households were found to be in the first rank, 1325 in the second rank, and 599 in the third rank or higher. As a result, the district development index was categorized into three groups: rank-1, rank-2, and rank-3 (index ≥ 3).

### Statistical analysis

Data were evaluated using IBM-SPSS version 26. The distribution of mother characteristics by region, residence, district developmental index, and risk awareness status were compared with Chi-square test. When there was a significant difference in a parameter in three district developmental index groups, adjusted residuals were calculated to find the group that makes the difference.

Multivariate binary logistic regression revealed determinants of presence of environmental risk perception in three models. Model 1 included mother-family-region-residence characteristics (region, residence, district developmental index, and mother related variables having a *p* value < 0.1 in bivariate analysis), Model 2 took dwelling environmental characteristics of home, and Model 3 covered both Model 1 and Model 2. Adjusted odds ratio (AOR) and 95% confidence intervals (CI) were calculated.

Analysis where a p value was below 0.05 were considered statistically significant.

## Results

### Household characteristics and demographic information

A total of 3549 mothers were interviewed and 3489 mothers with complete information were included in the study.

More than two thirds of mothers (69.7%) were from urban area. The frequency of working mothers was found higher in West Anatolia (*p* < 0.001, Table 1). The presence of more crowded families, defined as those with five or more people, and a higher number of children, defined as four or more, was observed in Central Anatolia (*p* < 0.001; Table 1).

**Table 1:** Household characteristics of mothers according to region, residence and district developmental index in West and Central Anatolia-Türkiye, 2008–2009, %^*^

		Region			Residence			District developmental index^**^			
	Overall	West Anatolia	Central Anatolia	*p*	Urban	Rural	p	Rank-1	Rank- 2	Rank-3	*p*
n	3489	2013	1476		2432	1057		1555	1325	599	
Mother’s age ≥ 35 yr	44.0	46.2	43.4	0.104	44.5	46.3	0.333	43.7	46.1	46.1	0.376
Father’s age ≥ 35 yr	74.1	74.9	73	0.220	74.4	73.4	0.548	74.9	73.9	72.7	0.593
Mothers’ education ≥ 8 yr	28.8	29.6	27.6	0.208	30.4	25.0	0.001	30.4^a^	31.5^a^	18.7^b^	< 0.001
Fathers’ education ≥ 8 yr	54.6	54.1	55.3	0.475	53.8	56.4	0.170	53.0	56.2	55.3	0.223
Working mother	9.8	11.2	7.8	< 0.001	10.6	7.8	0.008	10.1	10.6	7.3	0.077
Nuclear family	77.2	76.0	78.8	0.053	77.9	75.5	0.117	78.7	76.8	74.1	0.068
Household size ≥ 5 person	52.2	49.2	56.4	< 0.001	50.5	56.0	0.003	48.9^a^	53.4^b^	57.8^b^	0.001
Number of children											< 0.001
1	6.4	7.5	4.9	< 0.001	6.3	6.6	< 0.001	6.9	6.4	5.2	
2–3	72.3	76.2^a^	66.9^b^		74.7^a^	72.3^b^		74.1^a^	73.7^a^	64.3^b^	
≥ 4	21.3	16.3^a^	28.2^b^		19.0^a^	21.3^b^		19.0^a^	19.9^a^	30.6^b^	
Presence of any health problem in family	19.4	19.0	19.9	0.543	21.5	14.4	< 0.001	20.1^a^	22.0^a^	12.0^b^	< 0.001
Health problem in family members											
Asthma	5.7	5.2	6.3	0.152	6.5	3.7	0.001	6.0	6.2	3.7	0.067
Diabetes	2.5	2.3	2.8	0.297	2.8	1.9	0.118	2.5^ab^	3.2^a^	1.2^b^	0.035
Hypertension	4.7	4.6	4.7	0.939	4.9	4.1	0.287	4.6	5.4	3.0	0.064
Dermatitis	1.1	1.5	0.5	0.008	1.2	0.9	0.373	1.5^a^	0.9^ab^	0.3^b^	0.038
Malignancy	0.7	0.5	0.9	0.111	0.7	0.8	0.745	0.8	0.7	0.5	0.792
Goitre	5.7	5.7	5.7	0.972	6.5	3.9	0.003	6.2^a^	6.6^a^	2.3^b^	< 0.001

^*^column percentage

^**^there were 10 adresses as missing data in distinct developmental index

^ab^Values having different letters in the same row are different in their subgroup analysis, *p* < 0.05

The proportion of maternal education exceeding eight years was higher in urban areas compared to rural. Similarly, working mothers were found to be more prevalent in urban areas, as indicated by p-values of 0.001 and 0.008, respectively. Moreover, crowded household size was more frequently observed in rural areas compared to urban (*p* = 0.003, Table 1).

### Dwelling environmental characteristics

When the proximity to the factors that can be considered as environmental risk factors were questioned, dumpster areas, swamps or puddles, high voltage areas, transformer sites, and factory or industrial zones were stated as more prevalent in Central Anatolia. Construction sites, and rivers, streams are reported to be more common by mothers in West Anatolia (*p* < 0.05, Table 2).

**Table 2:** Dwelling environment characteristics of home according to region, residence, district developmental index, and health problem in West and Central Anatolia-Türkiye, 2008–2009,%^*^

		Region			Residence			District developmental index^**^				Any health problem in family		
Characteristics	Overall	West Anatolia	Central Anatolia	*p*	Urban	Rural	*p*	Rank-1	Rank- 2	Rank-3	*p*	none	presence	*p*
n	3489	2013	1476		2432	1057		1555	1325	599		2813	676	
Indoor smoking	48.4	49.0	47.4	0.348	49.7	45.2	0.015	49.1^a^	49.6^a^	43.6^b^	0.037	47.1	53.6	0.003
**Next to home**														
Main roads	48.7	49.8	47.2	0.126	53.5	37.6	< 0.01	58.2^a^	44.3^b^	33.2^c^	< 0.001	46.2	58.9	< 0.001
Agricultural areas	6.6	5.8	7.8	0.019	1.0	19.5	< 0.01	2.2^a^	2.9^a^	26.5^b^	< 0.001	7.2	4.3	0.007
Animal shelters	9.3	6.4	13.4	< 0.001	1.8	26.7	< 0.001	3.2^a^	4.5^a^	36.1^b^	< 0.001	9.9	7.1	0.026
Construction sites	9.7	11.1	7.9	0.001	11.7	5.2	< 0.001	8.9^a^	12.4^b^	6.2^c^	< 0.001	9.0	12.7	0.004
Dumpster areas	7.3	6.1	9.1	0.001	7.7	6.5	0.227	6.9	7.3	8.2	0.614	6.9	9.2	0.042
Swamps or puddles	2.3	1.3	3.6	< 0.001	1.9	3.0	0.048	1.5^a^	2.8^b^	3.0^b^	0.034	2.1	2.8	0.288
Rivers, streams	5	8.2	4.1	0.035	3.5	8.5	< 0.001	4.4^a^	3.2^a^	10.5^b^	< 0.001	4.8	6.2	0.122
High voltage areas	6.8	3.8	10.9	< 0.001	6.0	8.5	0.010	4.2^a^	6.7^b^	13.2^a^	< 0.001	6.5	7.8	0.228
Base station	18.8	18.2	19.6	0.313	20.3	15.3	0.001	21.7^a^	12.4^b^	24.9^a^	< 0.001	18.2	21.3	0.061
Transformer sites	17.6	15.4	20.7	< 0.001	19.2	14.1	< 0.001	19.7^a^	16.9^ab^	13.7^b^	0.003	16.4	22.6	< 0.001
Factory or industrial zones	6.6	5.5	8.3	0.001	7.6	4.4	< 0.001	11.4^a^	3.5^b^	1.2^c^	< 0.001	5.8	10.1	< 0.001

^*^column percentage

^**^There were 10 adresses as missing data in distinct developmental index

^abc^Values having different letters in the same row are different in their subgroup analysis, *p* < 0.05

Dwelling characteristics such as proximity to main roads, agricultural areas, animal shelters, swamps or puddles, rivers, streams, and high voltage areas were more commonly observed in rural areas (*p* < 0.05). Proximity to construction areas, base stations, transformer areas, factory or industrial zones, and smoking indoors were among the environmental features described more frequently by urban area dwellers (*p* < 0.05, Table 2).

When compared according to the district developmental index, all characteristics had statistically significant differences except living near dumpster areas (*p* < 0.05, Table 2).

The presence of a person with any health problem in the household was also related to the environmental characteristics of the place of residence. The presence of a smoker in the house, closeness to main roads, construction areas, garbage areas, and industrial areas were more frequent if there was a person with any disease in the house and this difference was significant (*p* < 0.05). On the other hand, the presence of a person with any disease was statistically less frequent in houses close to agricultural areas and animal shelters (*p* < 0.05, Table 2).

Indoor smoking was present in 48.0% (*n* = 1687) of the households; more in the urban residences, and district developmental index rank-1 and rank-2, family members having a disease than others (Table 2).

### Environmental risk knowledge of the mothers

Overall, 19.3% of the mothers stated that they did not know “what an environmental risk was”. Furthermore, 5.0% stated that there was no environmental risk. Those living in the Central Anatolia, living in rural areas and living in less developed areas were not aware of any risk factors, and it was statistically significant (*p* < 0.05). On average, one out of 5 mothers stated air pollution as an environmental risk (23%) which was the most frequent one, one out of 10 people stated municipal problems (12.4%), nonspecific environmental pollution (10.3%), radiation and electromagnetic field (9.9%) and accident risk (9.7%) as environmental risks (Table 3). Few mothers (< 1%) also stated disasters (*n* = 31), future anxiety (*n* = 33), and global warming (*n* = 24) as risks.

**Table 3:** Environmental risk knowledge of the mothers in West and Central Anatolia-Türkiye, 2008–2009, %^*^

		Region			Residence			District developmental index^**^				Any health problem in family		
	Overall	West Anatolia	Central Anatolia	*p*	Urban	Rural	*p*	Rank-1	Rank- 2	Rank-3	*p*	none	presence	*p*
n	3489	2013	1476		2432	1057		1555	1325	599		2813	676	
Air pollution	23.0	27.0	17.4	< 0.001	27	13.7	< 0.001	26.4^a^	24.9^a^	9.7^b^	< 0.001	21.6	28.4	< 0.001
Municipality, infrastuctural problems (construction, sewage system etc.)	12.4	14	10.2	0.001	12.5	12.2	0.808	12.6	11.3	14.2	0.197	11.7	15.2	0.013
Nonspecific environmental pollution	10.3	9.9	10.7	0.459	10.5	9.6	0.433	11.3	9.3	10.0	0.216	9.0	15.4	< 0.001
Radiation-EMF	9.9	9	11.2	0.033	10.4	8.9	0.182	11.3^a^	8.9^b^	8.2^b^	0.030	9.6	11.1	0.254
Accident risk	9.7	10.7	8.3	0.018	10.6	7.7	0.007	10.5^a^	11.4^a^	4.2^b^	< 0.001	8.5	14.6	< 0.001
Illness/death	7.1	4.5	10.6	< 0.001	7.5	6.1	0.120	6.8^a^	9.5^b^	2.3^c^	< 0.001	7.2	6.7	0.633
Safety	6.6	7.5	5.4	< 0.001	7.7	4	0.013	7.1	7.5	3.3	0.002	5.5	10.9	< 0.001
Food-water pollution	5.3	5.2	5.5	0.724	6.3	3.1	< 0.001	5.9	4.7	5.3	0.339	5.1	6.4	0.184
Communicable diseases	3.3	2.7	4.1	0.029	3.3	3.3	0.974	2.8	4.2	2.5	0.054	2.7	5.8	< 0.001
Noise	2.2	0.7	4.2	< 0.001	2.1	2.4	0.707	2.7	1.8	1.7	0.170	2.3	1.8	0.395
Chemical pollution	1.4	0.9	2	0.008	1.5	1.2	0.640	1.2	1.8	1.0	0.222	1.4	1.6	0.584
No danger	5.0	6.9	2.4	< 0.001	2.5	10.8	< 0.001	4.2^a^	6.6^b^	3.5^a^	0.002	5.2	4.1	0.261
Does not know	19.3	13.5	27.4	< 0.001	17.9	22.6	0.001	15.6^a^	19.5^b^	29.0^c^	< 0.001	20.5	14.3	< 0.001

*EMF* Electromagnetic field

^*^column percentage

^**^There were 10 adresses as missing data in distinct developmental index

^abc^Values having different letters in the same row are different in their subgroup analysis, *p* < 0.05

Mothers living in West Anatolia listed air pollution, municipal problems, accident risk, and safety issues as environmental risk factors more frequently (*p* < 0.05). On the other hand, mothers living in Central Anatolia stated general environmental pollution, radiation, illness-death, communicable diseases, noise, chemicals, and disasters as environmental risk factors at a higher rate (*p* < 0.05, Table 3).

The mothers living in the urban residences stated all environmental risk factors except noise more than the those living in rural residence. A statistically significant difference was found between rural and urban groups in terms of air pollution, accident risk, safety, and food-water pollution (*p* < 0.05, Table 3).

As the level of the district development status changed from rank-1 to rank-3, the frequency of mentioning air pollution, radiation, accident risk, and safety issues as environmental risks decreased significantly (*p* < 0.05). The environmental risks stated were changed with the presence of a family member with a chronic health problem. Air pollution, municipality problems, general environmental pollution, accident risk,  safety, and communicable diseases were mentioned more frequently by the families having a chronic health problem (*p* < 0.05, Table 3).

### The self-evaluation of the risks present in mothers’ environment

Of the total, 75.7% mentioned the existence of at least one risk factor in their environment. The number of mothers reporting risk factors was significantly higher in those living in West Anatolia and urban areas (*p* < 0.001). In less developed areas and in households with a family member with a health problem present, more mothers stated a risk factor, the difference was statistically significant (*p* < 0.05, Table 4). In addition, very few mothers reported the presence of illness/death (*n* = 25), chemicals (*n* = 9), disasters (*n* = 3), future anxiety (*n* = 1), and global warming (*n* = 1) in their neighborhood.

**Table 4:** The self-evaluation of the risks present in mothers’ environment in West and Central Anatolia-Türkiye, 2008–2009, %^*^

		Region			Residence			District developmental index^**^				Any health problem in family		
	Overall	West Anatolia	Central Anatolia	*p*	Urban	Rural	*p*	Rank-1	Rank- 2	Rank-3	*p*	none	presence	
n	3489	2013	1476		2432	1057		1555	1325	599		2813	676	
Air pollution	14.9	19.4	8.8	< 0.001	17.5	8.9	< 0.001	20.5^a^	13.9^b^	3.0^c^	< 0.001	13.7	19.8	< 0.001
Radiation-EMF	8.7	9	8.2	0.410	9.5	6.8	0.011	10.5^a^	6.3^b^	8.7^ab^	< 0.001	8.3	10.1	0.148
Municipality problems	7.5	9	5.4	< 0.001	8.3	5.6	0.004	8.4	6.9	6.8	0.249	6.6	11.2	< 0.001
Risk of accident	6.9	7.2	6.4	0.335	7.6	5.1	0.007	9.8^a^	6.2^b^	0.7^c^	< 0.001	5.8	11.1	< 0.001
Nonspecific environmental pollution	3.7	4.5	2.6	0.003	4.2	2.6	0.031	5.2^a^	2.3^b^	2.7^b^	< 0.001	3.0	6.5	< 0.001
Safety, stray animals	3.1	3.8	2	0.002	3.9	1.1	< 0.001	2.8^a^	4.2^a^	1.3^b^	0.003	2.6	5.0	0.001
Food-water pollution	2.9	3.6	1.9	0.003	3.7	1.0	< 0.001	4.1	2.2	1.5	0.001	3.0	2.4	0.362
Noise pollution	1.4	0.8	2.3	< 0.001	1.4	1.5	0.759	1.4	1.6	1.2	0.773	1.4	1.5	0.910
Communicable diseases	1.1	0.9	1.4	0.146	1.4	0.5	0.014	1.0^a^	1.6^b^	0.3^a^	0.049	0.9	1.9	0.027
Any risk stated	75.7	79.7	70.2	< 0.001	79.6	66.6	< 0.001	19.7^a^	26.1^b^	32.6^c^	< 0.001	74.3	81.5	< 0.001

*EMF* Electromagnetic field

^*^column percentage

^**^10 adresses were missing for distinct developmental index

^abc^Values having different letters in the same row are different in their subgroups' analysis, *p* < 0.05

Overall, the most frequently mentioned risk factor was air pollution (14.9%) and the second was radiation-EMF (8.7%). Both were more frequently mentioned in West Anatolia, urban areas, and more developed areas (*p* < 0.05). Municipality problems was in the 3^rd^ line (7.5%), and accident risk was in the 4^th^ line (6.9%) (Table 4).

Security (including stray animals) (3.1%), nonspecific environmental pollution (3.7%), food-water pollution (2.9%), and noise pollution (1.4%) were the other environmental risks stated by the mothers in their environment. Most of the risk factors were mentioned more in West Anatolia region and in urban areas. Only one person mentioned future anxiety and one person mentioned global warming as an environmental risk factor. When compared according to the district developmental index; risk of accidents, nonspecific pollution, stray animals and food-water pollution was mentioned more frequently in more developed areas (*p* < 0.05, Table 4).

As a note, there were 802 mothers who reported air pollution as an environmental risk. Of them, 5% stated that they smoked actively (*n* = 42). However, only 12 out of 1687 mothers with free indoor smoking reported “exposure to smoke” as a risk for their environment (0.7%) (*p* > 0.05).

### Conditions that related with risk awareness of mothers

Environmental risk perception status was evaluated according to mother characteristics. Regions, type of residences, environmental status of the house (living near highways or main roads, construction sites, dumping sites, swaps or puddles, stream or rivers, high voltage areas, base stations, transformer sites, factory or industrial centers), demographic characteristics (mothers’ and fathers’ education level, mothers’ working status, family type and household size) and the presence of an ill person in the family (any disease, asthma and goitre) associated with the perception of environmental risk at a statistically significant level (*p* < 0.05, Table 5). Surprisingly, tobacco exposure at home had not related to risk awareness status.

**Table 5:** Conditions that related with risk awareness of the mothers in West and Central Anatolia-Türkiye, 2008–2009

	*n*^a^	%^a^	*p*		*n*^a^	%^a^	*p*
**Mother age, yr**			0.594	**Residence**			< 0.001
< 35	1458	76.0		Urban	1936	79.6	
≥ 35	1182	75.2		Rural	704	66.6	
**Mother education, yr**			< 0.001	**NUTS region**			< 0.001
< 8	1814	73.0		West Anatolia	1604	79.7	
≥ 8	826	82.3		Central Anatolia	1036	70.2	
**Father education, yr**			< 0.001	**District development index**^**b**^			< 0.001
< 8	1113	70.9		Rank-1	1248	80.3^a^	
≥ 8	1506	79.8		Rank-2	979	73.9^b^	
**Moms' working status**			0.001	Rank-3	404	67.4^c^	
Working	2357	74.9		**Dwelling environmental characteristics**			
Not working	283	83.0		**Highway or main road**			
**Family type**			0.021	No	1232	68.8	< 0.001
Nuclear	2013	74.7		Yes	1408	82.9	
Other	627	78.8		**Agricultural areas**			0.408
**Household size**			0.012	No	2460	75.5	
< 5 person	1294	77.6		Yes	180	77.9	
≥ 5 person	1346	73.9		**Animal shelters**			0.821
**Number of children**			< 0.001	No	2395	75.7	
1	178	79.8^a^		Yes	245	75.2	
2–3	1965	77.9^a^		**Construction site**			
≥ 4	497	66.8^b^		No	2342	74.4	< 0.001
**Presence of disease in household members**				Yes	298	87.6	
**Any disease**			< 0.001	**Dumping site**			
No	2089	74.3		No	2416	74.7	< 0.001
Yes	551	81.5		Yes	224	87.5	
**Asthma**			0.006	**Swamp or puddle**			
No	2475	75.2		No	2568	75.3	0.001
Yes	165	83.8		Yes	72	91.1	
**DM**			0.690	**Stream or river**			
No	2575	75.7		No	2490	75.2	0.002
Yes	65	73.9		Yes	150	85.2	
**HT**			0.790	**High voltage areas**			
No	2516	75.6		No	2446	75.2	0.023
Yes	124	76.5		Yes	194	81.9	
**Goitre**			0.001	**Base station**			
No	2470	75.1		No	2053	72.4	< 0.001
Yes	170	85.9		Yes	587	89.6	
**Dermatit**			0.106	**Transformer sites**			
No	2607	75.5		No	2119	73.7	< 0.001
Yes	33	86.8		Yes	521	84.7	
**Cancer**			0.380	**Factory or industrial center**			
No	2620	75.6		No	2418	74.2	< 0.001
Yes	20	83.3		Yes	222	95.7	
**Smoking in home**			0.554				
No	1371	76.1		Overall	2640	75.7	
Yes	1269	75.2					

^a^n:cases having risk awareness in subgroup; %: row percentage for cases having risk awareness in subgroup

^b^10 adresses were missing for distinct developmental index

### Multiple logistic regression analysis regarding mothers’ environmental risk perception

Multiple logistic regression analysis revealed that the proportion of specifying at least one environmental risk factor is found to be more by 5.17 times for those who live in proximity to factory or industrial areas (95% CI 2.67–10.03), 2.63 times for those who live close to the swamps or puddles (95% CI 1.14–6.09), and 2.47 times for those who live near a base station (95% CI 1.86–3.29, *p* < 0.05, Table 6). As the maternal education increased, environmental risk factor awareness also increased by 1.44 times (95% CI 1.17–1.77). Compared to nuclear families, other family types have 1.29 times more environmental risks (95% CI 1.05–1.58), having a family member with a disease shows 1.28 times more risk (95% CI 1.02–1.61), dwelling near main roads displays 1.54 times more risk (95% CI 1.30–1.84), construction sites 1.50 (95% CI 1.05–2.14), or dumpster areas 1.87 times (95% CI 1.25–2.82).

**Table 6:** Determinants of mother’s environmental risk perception in West and Central Anatolia-Türkiye, 2008–2009, multivariate logistic regression analysis

	Model 1			Model 2			Model 3		
	AOR	95% CI	*p*	AOR	95% CI		AOR	95% CI	*p*
Residence; urban vs rural	1.95	1.59–2.40	< 0.001				1.86	1.49–2.32	< 0.001
Region; West Anatolia vs Central Anatolia	1.58	1.34–1.87	< 0.001				1.88	1.57–2.25	< 0.001
District developmental index			0.047						0.641
Rank-1 vs Rank-3	0.96	0.73–1.26	0.756				0.88	0.66–1.18	0.396
Rank-2 vs Rank-3	0.79	0.61–1.01	0.064				0.88	0.67–1.16	0.361
Mothers' education ≥ 8 vs < 8 years	1.56	1.28–1.91	< 0.001				1.44	1.17–1.77	0.001
Mothers’ working status; working vs stay-at-home	1.18	0.87–1.61	0.293				1.13	0.82–1.55	0.467
Other family types vs nuclear family	1.27	1.04–1.54	0.019				1.29	1.05–1.58	0.014
Children			0.003						0.009
Single child vs ≥ 4 children	1.36	0.93–1.99	0.113				1.31	0.88–1.95	0.178
2–3 children vs ≥ 4 children	1.39	1.15–1.68	0.001				1.36	1.12–1.65	0.002
Any disease in the household vs no disease	1.47	1.18–1.83	0.001				1.28	1.02–1.61	0.031
Dwelling near mainroads; presence vs absence				1.76	1.49–2.08	< 0.001	1.54	1.30–1.84	< 0.001
Dwelling near construction site; presence vs absence				1.78	1.26–2.52	0.001	1.50	1.05–2.14	0.024
Dwelling near dumpster areas; presence vs absence				1.69	1.14–2.52	0.009	1.87	1.25–2.82	0.003
Dwelling near puddles or swamps; presence vs absence				2.04	0.90–4.65	0.088	2.63	1.14–6.09	0.023
Dwelling near rivers, streams; presence vs absence				1.00	0.63–1.58	0.984	1.24	0.76–2.01	0.389
Dweeling near high voltage areas; presence vs absence				0.92	0.64–1.32	0.644	1.26	0.86–1.86	0.232
Dwelling near base station; presence vs absence				2.60	1.97–3.42	< 0.001	2.47	1.86–3.29	< 0.001
Dwelling near transformer site; presence vs absence				1.37	1.07–1.76	0.013	1.37	1.06–1.77	0.017
Dwelling near factories or industrial zones; presence vs absence				4.89	2.54–9.45	< 0.001	5.17	2.67–10.03	< 0.001
Constant	1.05		0.654	1.77		< 0.001	0.62		< 0.001

Model 1: mother-family-region-residence characteristics; Model 2: environmental characteristics of home; Model 3: both model 1 and model 2; *AOR* Adjusted odds ratio, *CI* confidence interval

## Discussion

In the current study, some of the key factors that can shape risk perception and tolerance of mothers were explored. Most mothers mentioned at least one risk factor indicating that the concept of environmental risk is prevalent among them. However, upon examining these risk factors individually, it is apparent that the importance of environmental exposure has not been fully comprehended yet. Air pollution is the most frequently mentioned environmental risk by the mothers especially by the urban residents, contrarily, awareness about chemical exposure, water pollution and general environmental pollution is much lower. This findings align with previous studies [29, 35–37]. A cross-sectional study from south-eastern France in 2017 showed that indoor/outdoor air quality and endocrine disruptors were the best-mastered topics for perinatal health professionals (962 participants) [38].

Although air pollution was the most frequent mentioned environmental risk in the present study, tobacco exposure was mentioned seldom as stated in the results. In the previous study, tobacco exposure was not associated with environmental pollution awareness [26]. There is a need to increase awareness of mothers on tobacco exposure and indoor air pollution especially for children. In a randomized controlled study conducted in Israel, it was observed that the smoking exposure perception of families was raised after they are informed about tobacco exposure and following air quality measurements was increased significantly [39]. In contrast to the present study, in another study conducted in the Sakarya province of Türkiye in 2014, 98.6% of participating parents identified smoking as an environmental risk factor [29]. It is important to note that this study employed a survey format with yes/no responses. The present study with open-ended questions, may provide a better reflection of risk perception with less bias. However, it is worth considering that regional factors could also contribute to these differences. Previous study in South Korea revealed that perceived educational needs of pregnant women included particulate matter (23.7%), electromagnetic waves (11.7%), instant food (food additives) (9.0%) and endocrine disrupting chemicals (8.3%) [40]. Therefore, there is a need for studies encompassing the entirety of Türkiye to further investigate this issue.

Present study showed higher risk perception among mothers living in urban residence, West Anatolia, educated 8 or more years. The study suggested that in West Anatolia, an increase in the education level of residents, as well as the proportion of working mothers were associated with a higher awareness of environmental risk factors in the region. A previous study suggested environmental pollution could be reduced by educating individuals and spreading information [41]. Similarly, education level and socioeconomic status were reported to constitute an important basis on the environmental risk awareness of families [15, 37, 42]. In addition, the socio-economic development level of the living environment were shown to be related with people's awareness [43]. This may be related to the level of education, as well as the fact that fewer people are included in the studies from regions with low socioeconomic status. On the other hand, the association between district developmental index and risk perception was disappeared in multivariate logistic regression analysis. This suggests that economic development alone might not significantly associated with risk perception. However, it is worth noting that when the level of economic development reaches a certain threshold, it can facilitate the implementation of new environmental remediation technologies and methods. These advanced technologies and methods have the potential to accelerate the restoration of the environment and mitigate environmental risks. In other words, economic development can create opportunities for adopting effective measures and practices to address environmental challenges and improve overall environmental conditions [44]. A Korean study showed age, perceived severity, and response efficacy of pregnant women affected pro-environmental behaviour [40].

In the present study, multivariate analysis showed that mothers living in near risky places like garbage dumps, factories, industrial areas, swamps, where environmental pollutants can be intense, have a higher risk awareness. As in line with this study, having a family member with asthma increases awareness and the support for precautionary measures [28]. There are many reports showing a positive correlation between environmental exposure and risk awareness [39, 45, 46]. However, it's important to note that risk awareness does not always translate into effective risk management. Some studies have found that despite having a high level of risk awareness, individuals may not necessarily engage in appropriate risk management practices [43, 47]. One study recruited in three heavily contaminated areas of Southern Italy revealed that risk perception index changed with studied site, besides, no association between risk perception and lifestyle during pregnancy was detected [48]. Another cross-sectional survey in the Amiata area on 2029 subjects aged 18–77 showed higher risk perception was among women and young people, associated with higher education [49]. This highlights the need for further research to better understand the risk management strategies employed by these individuals. By investigating the factors influencing risk perception and the barriers to effective risk management, future studies can contribute to the development of targeted interventions and policies aimed at reducing environmental risks and promoting healthier living environments. Also naturalist and social scientists’collaboration might play an important role in developing awareness about environmental risk factors [50]. Confirming our hypothesis, a previous quasi-experimental study in Korea showed that pregnant women having the pro-environmental prenatal education program including motivational education on eco-environmental protection had positive changes in environmental health perceptions and behaviors [51]. Similarly, the PREVED (PREgnancy, preVention, Endocrine Disruptors) project in France was developed to improve knowledge, to enhance risk perception, and to change exposure behavior by sharing know-how/experience in a positive non-alarmist approach [52].

### Strengths and limitations

This study is the most comprehensive study ever done in terms of being a survey including open-ended questions, the number of participants involved, inquiring relationship with their own health problems, and evaluating information about environmental risks in Türkiye. In a similar study environmental risk perception assessment was carried out in a smaller population in Adana, one province of Türkiye using a similar method [26], but the present study was done both by two NUTS regions and by using the district development index in a much larger population.

The study's strength lies in its differentiation according to NUTS regions and the inclusion of each province based on the rural–urban population proportion, which enhances its representativeness. In parallel, the use of random and impartial sample selection from the population records also minimized the possibility of selection bias. This study has the power to represent the sampled West Anatolia and Central Anatolia. There is a need for more comprehensive studies that can represent the whole of Türkiye.

In the studies, where the data were collected via self-statements, there is always a possibility that respondents may not fully express their true thoughts or may intentionally hide certain information. This can introduce a bias in the data collected and affect the accuracy of the findings. Moreover, since the present study employed a cross-sectional design, it is important to note that it does not establish a causal relationship between the factors examined. Due to the large sample size, some unrelated factors may seem correlated.

The temporal gap between the execution of the study in 2008–2009 and the subsequent submission of the article in the current year (2023) indeed raises a pertinent consideration regarding the potential impact of this delay on the validity and relevance of the study's results and findings. The extended interval between data collection and article submission could introduce certain limitations, such as changes in technological advancements, policy shifts, or other external factors that might have influenced the context in which the study's outcomes are situated. However, it's important to recognize that certain types of research possess enduring relevance beyond their initial timeframe. Moreover, in our research, where outcomes are significantly influenced by socio-economic impacts, certain fundamental patterns and relationships may remain relatively stable over time, even as specific contextual elements evolve. Being the first study on this subject in the national field is also important in order to observe the changes in environmental pollutants in the society and to make perceptual awareness plans in the society.

## Conclusion

In order to take precautions against environmental risk factors and influence policy decisions, it is crucial to increase environmental risk awareness of society. The present study showed that characteristics of the area, in which mothers reside affects environmental risk factor comprehension. It is very important to create environmental awareness proactively in order to protect the well-being of the children, society and environmental health. Health authorities need to prioritize raise public awareness on this issue. There is a need for a more comprehensive evaluation with studies to be carried out by taking samples from all segments of the society in the future.

## Data Availability

For access to the files, please send an e-mail request to siyalcin@hacettepe.edu.tr.
